# Supplementary material for: Associations of treated and untreated human papillomavirus infection with preterm delivery and neonatal mortality: A Swedish population-based study
Source: PLoS Med. 2021 May 10;18(5):e1003641. doi: 10.1371/journal.pmed.1003641 (PMC8143418; doi:10.1371/journal.pmed.1003641)
Supplement: S1 STROBE Checklist — (DOC) [file pmed.1003641.s001.doc]

**STROBE Checklist**

STROBE Statement—checklist of items that should be included in reports of observational studies

|  | Item No | Recommendation | Section, Paragraph No. | Text from manuscript or authors comment |
| --- | --- | --- | --- | --- |
| **Title and abstract** | 1 | (*a*) Indicate the study’s design with a commonly used term in the title or the abstract | **Title**  **Abstract;** | *“Associations of treated and untreated human papillomavirus infection with preterm delivery and neonatal mortality: A Swedish population-based study”*  *“This was a retrospective population-based register study”.* |
|  |  | (*b*) Provide in the abstract an informative and balanced summary of what was done and what was found | **Abstract**  -Methods and findings  **Abstract**-Conclusion | *“Study groups were defined based on cervical HPV-tests, cytology and histology, as registered in the Swedish National Cervical Screening Registry.” “Study groups were compared by logistic regression and comparisons were adjusted for socioeconomic and health-related confounders”.*  *“In this study, we found that HPV infection in conjunction with pregnancy was associated with PTD, pPROM, PROM and neonatal mortality. Previous treatment for CIN was associated with even greater risks for PTD and pPROM and was also associated with PROM, neonatal mortality and maternal and neonatal infectious complications”.* |
| Introduction | | |  |  |
| Background/rationale | 2 | Explain the scientific background and rationale for the investigation being reported | **Introduction**  Background  Para 1  Para2  Para 3  Para 4 | *” Unfortunately, surgical excision to treat CIN has been associated with an increased risk of preterm delivery (PTD), preterm preleabour rupture of membranes (pPROM), conditions requiring neonatal intensive care and with neonatal mortality in subsequent pregnancies. PTD, birth before 37 weeks of gestation, is an enormous global obstetric and societal problem, as it is the main cause of mortality in children under the age of five, as well as of short- and long-term morbidity. “*  *“The mechanism behind excisional treatment increasing the risk of PTD remains unclear”*  *“there is limited knowledge concerning whether PTD after excisional treatment is associated with infectious complications”*  *“It is thus unknown whether a HPV infection can cause PTD or other adverse obstetric outcome”.*  *” Women with untreated CIN also seem to have an increased risk of PTD, but it has not been established whether this risk is attributable to HPV infection during pregnancy”.* |
| Objectives | 3 | State specific objectives, including any prespecified hypotheses | **Introduction**  Background  Para 4 | *“In this large Swedish national population-based study we aimed to investigate whether HPV infection in conjunction with pregnancy is associated with an increased risk of PTD and other adverse obstetric and neonatal outcomes. We also aimed to study the association between previous treatment for CIN and PTD and other adverse obstetric and neonatal outcomes, with special focus on infectious complications at birth”.* |
| Methods | | |  |  |
| Study design | 4 | Present key elements of study design early in the paper | **Methods and Material**  Data sources, Para1 | “*This study is a retrospective population-based study using data from different Swedish health and quality registers”.* |
| Setting | 5 | Describe the setting, locations, and relevant dates, including periods of recruitment, exposure, follow-up, and data collection | **Methods and Material,** Data sources  Para 1  Study population  Para 1  **Figure 1**  **Methods and Material**  Exposures  **Supporting Information**  S2 Tables  S3 Tables | *“This study is a retrospective population-based study using data from different Swedish health and quality registers”*  *“All women with singleton births between 1999 and 2016 were identified in the MBR”.*  *“Flowchart of the study population”*  *“A delivery registered in the MBR was eligible for inclusion in a study group if the woman fulfilled the critera for exposure, i.e.results of cervical HPV tests registered in the NKCx 2007-2016, cervical cytology and histology registered in the NKCx 1978-2016 and/or histological diagnoses registered in the Swedish Cancer Register. For exact classification of study groups, see S2 Table.”*  *“Definition of study groups”*  *“Classification of Cervical Cytology and Histology “* |
| Participants | 6 | (*a*) *Cohort study*—Give the eligibility criteria, and the sources and methods of selection of participants.  Describe methods of follow-up  *Case-control study*—Give the eligibility criteria, and the sources and methods of case ascertainment and control selection. Give the rationale for the choice of cases and controls  *Cross-sectional study*—Give the eligibility criteria, and the sources and methods of selection of participants | **Methods and Material,**  Study population  Para 1  **Supporting information**  S1 Table  **Methods and Material,**  Exposures  Para 1  **Supporting Information**  S2 Table | *“All women with singleton births between 1999 and 2016 were identified in the MBR. Women with a history of chronic inflammatory disease, human immunodeficiency virus infection or organ transplantation were excluded”*  *“Diagnosis codes, according to the International Statistical Classification of Diseases 10 (ICD-10), registered in the Swedish Medical Birth Register, leading to exclusion”.*  *“A Reference group was defined, consisting of women who had a history of exclusively normal cervical cytology results until end of the study period, as well as at least one sample taken in the three years preceding the included delivery (n=338,109).*  *The exposure groups were defined as follows:*   1. *HPV infection group: Women with presumed HPV infection during pregnancy, based on abnormal cervical cytology or positive HPV test recorded within six months prior to conception or during pregnancy. Two partly overlapping groups were defined:*   *1a) HPV infection (cytology): abnormal cytology (n=11,727)*  *1b) HPV infection (HPV test): positive cervical HPV test (n=2,550)*   1. *Subsequent CIN2+ group: at presumed risk of persistent HPV infection based on a histological diagnosis of CIN2-3, adenocarcinoma in situ (AIS) or cervical cancer any time after delivery (n=33,760).* 2. *Treated group: histologically diagnosed CIN3 before conception. Since there is no national Swedish register covering treatment for CIN, CIN3 was used as a proxy for treatment as these women are always treated in Sweden, a policy that has been consistent over the study period (n=23,185).*   *In order to avoid women previously treated for CIN being included in the HPV infection groups or the Subsequent CIN2+ group, women with histologically diagnosed CIN1 more than once or with CIN2+ before the pertinent delivery were excluded*  *from these groups. “*  *“Definition of study groups“* |
|  |  | (*b*) *Cohort study*—For matched studies, give matching criteria and number of exposed and unexposed  *Case-control study*—For matched studies, give matching criteria and the number of controls per case |  |  |
| Variables | 7 | Clearly define all outcomes, exposures, predictors, potential confounders, and effect modifiers. Give diagnostic criteria, if applicable | **Methods and Material**  Exposure  Para 1  **Supporting Information**  S2 Table  S3 Table  **Methods and Material** Outcomes  Para 1  Para 2  Para 3  **Supporting Information**  S4 Table  **Methods and Material**  Background Variables  Para 1  Statistical Analyses  Para 4  **Results**  Caracterisitics of the study population  Table 1 | Comment; Please see Participants=#6 for study groups  *“Definition of study groups“*  *“Classification of Cervical Cytology and Histology”*  *“The primary outcome was PTD at 22-37 weeks of gestation (154-258 days). Gestational age was retrieved from the MBR using the best estimate, i.e. ultrasound determination when available and last menstrual period or estimation of gestational age at delivery ward in the remaining cases. Subanalyses for early PTD (22-34 weeks or 154-237 days of gestation) and for very early PTD (22-28 weeks or 154-195 days of gestation) were performed“*  *“Secondary outcomes were pPROM (determined according to ICD-10 codes in the MBR) and spontaneous PTD (defined as pPROM or preterm labour, excluding deliveries that started with induction or caesarean section, according to ICD-10 codes and MBR parameters) “*  *“Further studied outcomes comprised PROM in term pregnancies, chorioamnionitis, intrapartum fever, neonatal sepsis, Apgar score < 7 at five minutes, intrauterine fetal death, neonatal mortality (1-28 days) and small for gestational age (SGA) defined as birthweight less than -2 standard deviations (SD) according to the Swedish reference curves”*  *“Outcome definition based on diagnosis codes according to the International Classification of Diseases 10 (ICD-10) in the Swedish Medical Birth Register.”*  *“The following background variables were retrieved from the MBR and from SCB registers: age at delivery, body mass index (BMI), smoking before and during pregnancy, marital status, education level at delivery, employment at delivery, disposable household income three years preceding delivery, country of birth, parity, infant’s sex and assisted reproduction, as well as chronic renal disease, diabetes, epilepsy and chronic hypertension as reported in antenatal care records“*  *“The multivariable analyses were adjusted for potential confounding factors, including background variables with an uneven distribution between the groups, i.e. year of delivery (1999-2001, 2002-2004, 2005-2007, 2008-2010, 2011-2013, 2014-2016), maternal age (<21, 21-25, 26-30, 31-35, 36-40, >40), parity (0,1,2,3,>3), BMI (underweight (<18.5), normal weight (18.5-24.9), overweight (25-29.9), obese (≥30), missing), marital status (cohabiting, single, other, missing), country of birth (Sweden, Europe, Asia, America/Oceania, Africa, other/unknown), infant’s sex (boy/girl), smoking (never, before pregnancy, in early pregnancy, in the third trimester, missing), highest disposable household income three years preceding delivery (population divided into tertiles for every year), education level (primary, secondary, post-secondary <3years, post-secondary ≥3 years, missing) and assisted reproduction (yes/no).“*  *“Demographics and clinical characteristics in the study groups.”* |
| Data sources/ measurement | 8* | For each variable of interest, give sources of data and details of methods of assessment (measurement). Describe comparability of assessment methods if there is more than one group | **Methods and Material**  Data Sources  Study Population  Figure 1  Exposure  Outcomes  Background Variables  **S1-4 Tables** | *“data from different Swedish health and quality registers”*  Exposure variables came from the Swedish National Cervical Screening Registry and The Swedish Cancer Register (S2-3 Tables).  Outcomes were constructed from existing variables in the mandatory Swedish Medical Birth Register (MBR) including ICD codes reported in the MBR (S4 table).  See also #7. |
| Bias | 9 | Describe any efforts to address potential sources of bias | **Methods and Material**  Study population  Para 1  **Supporting information**  S1 Table | *“singleton births”*  *“Women with a history of chronic inflammatory disease, human immunodeficiency virus infection or organ transplantation were excluded”*  *“Diagnosis codes, according to the International Statistical Classification of Diseases 10 (ICD-10), registered in the Swedish Medical Birth Register, leading to exclusion.”*  Comment;  Chronic inflammatory disease, HIV infection, earlier organ transplantation and related treatment and multiple pregnancy confers an increased risk for adverse obstetrical outcome. To better study the effect of HPV infection and earlier treatment for CIN we therefore decided to exclude them. |
| Study size | 10 | Explain how the study size was arrived at | **Methods and Material**  Study population  Para 1  **Figure 1** | *“All women with singleton births between 1999 and 2016 were identified in the MBR. Women with a history of chronic inflammatory disease, human immunodeficiency virus infection or organ transplantation were excluded (see S1 Table for the International Statistical Classification of Disease (ICD) codes leading to exclusion), leaving a study population comprising 1,787,842 deliveries in 1,044,023 women. Of these, 400,583 women had at least one delivery that fulfilled the criteria for inclusion in a study group”*  *“Flowchart of the study population”* |
| Quantitative variables | 11 | Explain how quantitative variables were handled in the analyses. If applicable, describe which groupings were chosen and why | **Methods and Material**  Outcomes  Para 1  Para 3  Statistical analyses  Para 3 | “*PTD at 22-37 weeks of gestation (154-258 days)” “early PTD (22-34 weeks or 154-237 days of gestation) and for very early PTD (22-28 weeks or 154-195 days of gestation)”*  *“neonatal mortality (1-28 days)”*  *“small for gestational age (SGA) defined as birthweight less than -2 standard deviations (SD) according to the Swedish reference curves “*  Comment; Grouping of potential confounding factors for example; “*BMI (underweight (<18.5), normal weight (18.5-24.9), overweight (25-29.9), obese (≥30), missing)”.* |
| Statistical methods | 12 | (*a*) Describe all statistical methods, including those used to control for confounding | **Methods and Material**  Statistical analyses  Para 1  Para 3 | “*The study groups were compared concerning obstetric and neonatal outcomes by unadjusted and adjusted logistic regression analysis”.*  “*The multivariable analyses were adjusted for potential confounding factors, including background variables with an uneven distribution between the groups…”* |
|  |  | (*b*) Describe any methods used to examine subgroups and interactions | **Methods and Material**  Statistical analyses  Para 2  Para 4  Para 5 | *“the Subsequent CIN2+ group was compared to the Reference group after stratification for interval (0-3 years or >3 years) to diagnosis of CIN2+”.*  *“Women with a delivery included in the Subsequent CIN2+ group, followed by a delivery included in the Treated group, were included in paired analyses with conditional logistic regression analysis”.*  *“In order to study the implication of infectious complications, the Treated group was compared with the Reference group regarding chorioamnionitis and neonatal sepsis, with adjusted logistic regression after stratification for PTD. The Treated group was also compared with the Reference group regarding chorioamnionitis and neonatal sepsis in PTD cases, after stratification for pPROM and also adjusted for gestational age.”*  “*Subgroup analysis comparing neonatal mortality in the Treated and Reference groups was also performed, after stratification for infectious complications (chorioamnionitis and/or neonatal sepsis) and also adjusted for gestational age.”* |
|  |  | (*c*) Explain how missing data were addressed | **Figure 1**  Table 1  **Methods and Material**  Statistical analyses  Para 3  **Results**  Small for gestational age and Apgar score | *“Flowchart of the study population”*  Comment; women missing gestational age were excluded from the study.  *“Demographics and clinical characteristics in the study groups”.* Comment; Missing background data is shown in Table 1.  Comment; If data for a variable adjusted for in the multivariable regression analyses were missing for any woman a separate category for missing variables were constructed and used,  “*marital status (cohabiting, single, other,* ***missing****),”*  *“Data was missing for 4,028 deliveries (0.2%)”.* Comment; For the outcome SGA some women had missing data- the number is reported in Results and in Tables 2-5. |
|  |  | (*d*) *Cohort study*—If applicable, explain how loss to follow-up was addressed  *Case-control study*—If applicable, explain how matching of cases and controls was addressed  *Cross-sectional study*—If applicable, describe analytical methods *taking account of sampling strategy* | **Results**  Table 1 | Comment; Our definition of study groups resulted in different background factors – see Table 1.  We adjusted for those in the logistic regression analyses. |
|  |  | (*e*) Describe any sensitivity analyses | **Methods and Material**  Statistical Analyses  Para 2  **Results**  Table 3  **Supporting information**  S6 Table  **Methods and Material**  Statistical Analyses  Para 2  **Supporting information**  S8 Table | *Comment;* The Subsequent CIN2+ groups <=3 years and >3 years to diagnosis were compared with unadjusted and adjusted logistic regression.  *“Obstetric and neonatal outcomes in the Subsequent CIN2+ group, compared with the Reference group, stratified for interval from delivery to CIN2+ diagnosis, adjusted multivariable logistic regression analyses.”*  *“Obstetric and neonatal outcome in the Subsequent CIN2+ group, compared with the Reference group, stratified for interval from delivery to CIN2+ diagnosis, univariable logistic regression analyses.”*  Comment; Abnormal cytology was used as a proxy for HPV infection and in order to test if cytology was a valid substitute for a positive HPV test, the two HPV infection groups were compared to each other with unadjusted and adjusted logistic regression analyses.  *“Outcomes in the HPV infection (cytology) group, compared to the HPV infection (HPV test) group, univariblee and multi-variable logistic regression analyses, deliveries 2007-2016. “* |

| Results | | |  |  |
| --- | --- | --- | --- | --- |
| Participants | 13* | (a) Report numbers of individuals at each stage of study—eg numbers potentially eligible, examined for eligibility, confirmed eligible, included in the study, completing follow-up, and analysed | **Methods and Material**  Study population  **Results**  Figure 1 | *“Flowchart of the study population“* |
|  |  | (b) Give reasons for non-participation at each stage | **Methods and Material**  **Figure 1**  Study population | *“Flowchart of the study population”* |
|  |  | (c) Consider use of a flow diagram | **Figure 1** | *“Flowchart of the study population”* |
| Descriptive data | 14* | (a) Give characteristics of study participants (eg demographic, clinical, social) and information on exposures and potential confounders | **Results**  Table 1 | *“Demographics and clinical characteristics in the study groups”.* |
|  |  | (b) Indicate number of participants with missing data for each variable of interest | **Results**  Table 1 | *“Demographics and clinical characteristics in the study groups.”* |
|  |  | (c) *Cohort study*—Summarise follow-up time (eg, average and total amount) |  |  |
| Outcome data | 15* | ***Cohort study*—Report numbers of outcome events or summary measures over time** | **Results** | Comment; for every outcome the result for the whole dataset is also reported. |
|  |  | ***Case-control study—*Report numbers in each exposure category, or summary measures of exposure** |  |  |
|  |  | ***Cross-sectional study—*Report numbers of outcome events or summary measures** |  |  |
| Main results | 16 | **(*a*) Give unadjusted estimates and, if applicable, confounder-adjusted estimates and their precision (eg, 95% confidence interval). Make clear which confounders were adjusted for and why they were included** | **Methods and Material**  Statistical Analyses  Para 3  **Results**  Tables 2-5 and  **Supporting information**  S8 Table  **Figures 2a, 2b, 3**  **Results**  Table 5  **Supporting information**  S5-8 Tables | Comment; Description of adjustments  Comment; Adjusted analyses is described in text under every outcome.  Description about what was adjusted for is under every table.    Adjusted analyses.  Comment; Unadjusted analyses |
|  |  | (*b*) Report category boundaries when continuous variables were categorized | **Methods and Material**  Outcomes  Para 1 and 3 | Comment; boundaries for PTD, early PTD, very early PTD, neonatal mortality and SGA. |
|  |  | (*c*) If relevant, consider translating estimates of relative risk into absolute risk for a meaningful time period |  |  |
| Other analyses | 17 | Report other analyses done—eg analyses of subgroups and interactions, and sensitivity analyses | **Methods and Material**  Statistical Analyses  Para 2  **Results**  Table 3  and **Supporting information**  S6 Table  **Methods and Material**  Statistical Analyses  Para 4  And Para 5  **Results**  Infectious Complications  And  Neonatal Mortality  **Methods and Material**  Statistical Analyses  Para 2  **Results**  Paired analyses  **Table 5**  **Methods and Material**  Statistical Analyses  Para 2  **Supporting information**  S8Table  **Methods and Material**  Statistical Analyses  Para 2  **Results**  Table 3  **Supporting information**  S6Table | Comment; Subgroups of Subsequent CIN2+ <=3 years after delivery or > 3 years after delivery  *“Obstetric and neonatal outcomes in the Subsequent CIN2+ group, compared with the Reference group, stratified for interval from delivery to CIN2+ diagnosis, adjusted multivariable logistic regression analyses.”*  *“Obstetric and neonatal outcome in the Subsequent CIN2+ group, compared with the Reference group, stratified for interval from delivery to CIN2+ diagnosis, univariable logistic regression analyses.”*  Comment; The treated group was compared with the reference group for infectious complications after stratification for PTD and for pPROM.  The treated group and the reference group were also compared for neonatal mortality after stratification for infectious complications or not (chorioamnionitis/neonatal sepsis). Differences in OR between strata were analysed by an interaction term between the strata and the group in logistic regression  Comment; Paired analyses  *“Obstetric and neonatal outcomes in paired analyses of a woman´s last delivery before treatment, compared to the first delivery after treatment; conditional logistic regression analysis, n=4,680 women”*  Comment; Sensitivity analyses; the two HPV infection groups were compared to each other with adjusted logistic regression, S9 Table;  *“Outcomes in the HPV infection (cytology) group, compared to the HPV infection (HPV test) group, univariable and multivariaböe logistic regression analyses, deliveries 2007-2016.”*  Comment: the Subsequent CIN2+ groups was split into; <=3 years and >3 years to diagnosis and subgroups were compared to the reference group with unadjusted and adjusted logistic regression. These subgroups were also compared to each other. |
| Discussion | | |  |  |
| Key results | 18 | Summarise key results with reference to study objectives | **Discussion**  Main findings  **Conclusion** | “*In this population-based study, women with HPV infection in conjunction with pregnancy had an increased risk of PTD, spontaneous PTD, pPROM, PROM and neonatal mortality. An even higher risk of PTD, spontaneous PTD and pPROM was found in women with previous treatment for CIN. Previous treatment was also associated with PROM, neonatal mortality and maternal and neonatal infectious complications”.* |
| Limitations | 19 | Discuss limitations of the study, taking into account sources of potential bias or imprecision. Discuss both direction and magnitude of any potential bias | **Discussion**  Study strengths and limitations | *“As this is an observational study, causality cannot be established and residual confounding cannot be ruled out despite thorough adjustments”*  *“A small minority of HPV tests might have been positive due to exclusive detection of low-risk strains”” abnormal cytology was used as a proxy for HPV infection as in earlier studies” Positive HPV tests/cytology up to six months before conception were included in the HPV infection groups, and some infections might have resolved before pregnancy. However, such misclassification would have attenuated a true effect. We did not require a negative HPV test in the Reference group and some women might have had an undetected HPV infection. However, such misclassification would have attenuated the associations found between the exposure groups compared to the Reference group. Since no national treatment data are available, CIN3 was used as a proxy for treatment; all women treated for high-grade lesions are thus not included in the Treated group. For the paired analyses maternal age and parity were not possible to adjust for and these results must thus be interpreted with caution”* |
| Interpretation | 20 | Give a cautious overall interpretation of results considering objectives, limitations, multiplicity of analyses, results from similar studies, and other relevant evidence | **Discussion** Comparision with previous studies and interpretation |  |
| Generalisability | 21 | Discuss the generalisability (external validity) of the study results | **Discussion**  Study strengths and limitations | Comment; This study is a large national population-based study. The surgical treatment methods, the HPV tests used and the SNOMED codes for definition of exposure in this study are used world-wide and so are also the definitions of outcomes in this study (based on ICD-10). This support generalizability. |
| Other information | | |  |  |
| Funding | 22 | Give the source of funding and the role of the funders for the present study and, if applicable, for the original study on which the present article is based |  | Funding: JW has received research grants from Østfold Hospital Trust, Norway (nr 16/04196), and Hjalmar Svenssons forskningsfond, Sweden (nr HJSV2020079). VS has received research grants from Wilhelm och Martina Lundgren Vetenskapsfond (2016-1005) and Fru Mary von Sydows, född Wijk, donationsfond (nr 1016). “The funders had no role in study design, data collection and analysis, decision to publish, or preparation of the manuscript.” |

*Give information separately for cases and controls in case-control studies and, if applicable, for exposed and unexposed groups in cohort and cross-sectional studies.

**Note:** An Explanation and Elaboration article discusses each checklist item and gives methodological background and published examples of transparent reporting. The STROBE checklist is best used in conjunction with this article (freely available on the Web sites of PLoS Medicine at http://www.plosmedicine.org/, Annals of Internal Medicine at http://www.annals.org/, and Epidemiology at http://www.epidem.com/). Information on the STROBE Initiative is available at [www.strobe-statement.org](http://www.strobe-statement.org/).
