# Supplementary material for: Associations of treated and untreated human papillomavirus infection with preterm delivery and neonatal mortality: A Swedish population-based study
Source: PLoS Med. 2021 May 10;18(5):e1003641. doi: 10.1371/journal.pmed.1003641 (PMC8143418; doi:10.1371/journal.pmed.1003641)
Supplement: S1 Table — (DOC) [file pmed.1003641.s002.doc]

**S1 Table. Diagnosis codes, according to the International Statistical Classification of Diseases 10 (ICD-10), registered in the Swedish Medical Birth Register, leading to exclusion.**

| **Inflammatory arthritis and systemic inflammatory disease** |
| --- |
| M05, M05.0, M05.1, M05.2, M05.3, M05.8, M05.8A, M05.8B, M05.8C. M058D, M058F, M058G, M058H, M05.8L, M05.8M, M05.8N, M05.8X, M05.9, M05.9L, M05.9M, M05.9N  M06.0, M06.0L, M060M, M06.0N, M06.1, M06.2, M06.3, M06.4, M06.8, M06.8L, M06.8L, M06.8M, M06.9, M06.9A, M06.9B, M06.9C, M06.9D, M06.9F, M06.9G, M06.9H, M06.9X  M08.0, M08.0B, M08.0C, M08.0D, M08.0F, M08.0G, M08.0H, M08.0X, M08.1, M08.2, M08.2A, M08.2B, M08.3, M08.4, M08.4A, M08.4B, M08.8, M08.9  M30.0, M30.1, M30.2, M30.3, M30.8  M31.0, M31.1, M31.3, M31.4, M31.5, M31.6, M31.7, M31.8  M32, M32.0, M32.1, M32.8, M32.8A, M32.8B, M32.8C, M32.8D, M32.8W, M32.9  M33.0, M33.1, M33.2, M33.9  M34.0, M34.1, M34.2, M34.8, M34.9  M35.0, M35.0A, M35.0B, M35.1, M35.2, M35.3  K75.4 |
| **Inflammatory bowel disease** |
| K50.0, K50.1, K50.8, K50.9  K51, K51.0, K51.2, K51.3, K51.4, K51.5, K51.8, K51.9 |
| **Organ transplantation** |
| Z94.0, Z94.1, Z94.2, Z94.3, Z94.4, Z94.6, Z94.8, Z94.9 |
| **Human immunodeficiency virus (HIV) infection** |
| O987  B20, B20.1, B204  B22  B23.1, B23.2, B23.8  B24 |

Abbreviations: ICD=International Statistical Classification of Diseases and Related Health Problems; MBR=Medical Birth Register
