# Supplementary material for: Associations of treated and untreated human papillomavirus infection with preterm delivery and neonatal mortality: A Swedish population-based study
Source: PLoS Med. 2021 May 10;18(5):e1003641. doi: 10.1371/journal.pmed.1003641 (PMC8143418; doi:10.1371/journal.pmed.1003641)
Supplement: S2 Table — (DOC) [file pmed.1003641.s003.doc]

**S2 Table. Definition of study groups.**

| **Study group** | **Exposures as criteria for inclusion^1^** | **Additional criteria for inclusion^1^** | **Comment** |
| --- | --- | --- | --- |
| Reference group | Only normal cervical cytology samples  AND  Samples taken at least every fifth year from age 23 years^2^ until end of study period or age 45 years  AND  At least one sample taken in the 3 years preceding the included delivery | No positive cervical HPV test within 6 months prior to conception or during the pregnancy | This group consists of women who have participated in the Swedish cervical screening program and have no recorded abnormal cervical cytology sample. |
| Exposure Groups | | | |
| 1a) HPV infection (cytology) | At least one abnormal cervical cytology sample within 6 months prior to conception or during the pregnancy, i.e.  low-grade (ASCU-S, CIN1) or  high-grade (ASC-H, CIN2, CIN3, squamous cell cancer, AGUS and AIS) | Prior to delivery:  No cervical biopsies  OR  Only normal biopsies  OR  Only one biopsy with histological diagnosis of CIN1 | This group consists of women with a presumed HPV infection during pregnancy, based on an abnormal cervical cytology test in close connection to or during pregnancy.  No treatment for CIN before delivery is presumed, since there is no histologically diagnosed CIN2+ in the database. |
| 1b) HPV infection (HPV test) | A positive cervical HPV test taken within 6 months prior to conception or during the pregnancy | Prior to delivery:  No cervical biopsies  OR  Only normal biopsies  OR  Only one biopsy with histological diagnosis of CIN1 | This group consists of women with presumed HPV infection during pregnancy, based on a positive HPV test in close connection to or during pregnancy.  No treatment for CIN before delivery is presumed, since there is no histologically diagnosed CIN2+ in the database. |
| 2) Subsequent CIN2+ | After the included delivery, the women were histologically diagnosed with CIN2, CIN3, cervical squamous cell cancer, AIS or cervical adenocarcinoma | Prior to delivery:  No cervical biopsies  OR  Only normal biopsies  OR  Only one biopsy with histological diagnosis of CIN1 | No treatment for CIN before delivery is presumed, since there is no histologically diagnosed CIN2+ in the database.  Hypothesis: for some reason, these women have an increased risk of persistent HPV infection since they develop CIN2+, cancer or AIS later in life. |
| 3) Treated | Histologically diagnosed CIN3 before conception |  | These women are presumed to have been treated before pregnancy. |

^1^ Based on cytology and histology in the Swedish National Cervical Screening Registry and/or the Swedish Cancer Register

^2^ Women in Sweden are invited for cervical screening every third year, beginning from 23 years of age.

AIS, adenocarcinoma in situ; CIN, cervical intraepithelial neoplasia; HPV, human papillomavirus
