# Supplementary material for: Associations of treated and untreated human papillomavirus infection with preterm delivery and neonatal mortality: A Swedish population-based study
Source: PLoS Med. 2021 May 10;18(5):e1003641. doi: 10.1371/journal.pmed.1003641 (PMC8143418; doi:10.1371/journal.pmed.1003641)
Supplement: S3 Table — (DOC) [file pmed.1003641.s004.doc]

**S3 Table. Classification of cervical cytology and histology.**

|  | **Squamous cell classification** | | | | |
| --- | --- | --- | --- | --- | --- |
|  |  |  | SNOMED^1^  From Swedish National Cervical Screening Registry | | Swedish Cancer Register |
|  |  |  | Cytology | Histology | Histology* |
| Normal |  | Normal cytology | M00110 |  |  |
| Low- grade | ASCU-S | Atypical squamous cells of undetermined significance | M69710 |  |  |
|  | CIN1 | Mild atypia/Cervical intraepithelial neoplasia grade1 | M74006 | M74006 |  |
| High- grade | ASC-H | Atypical squamous cells -cannot exclude high-grade squamous intraepithelial lesion | M69719 | M69719 |  |
|  | CIN2 | HSIL/Moderate atypia | M74007 | M74007 |  |
|  | CIN3/CIS | HSIL/Severe atypia | M80702 | M80702 | 144/b |
|  |  | Squamous cell carcinoma | M80703 | M80703 | 146 |

|  | **Glandular cell classification** | | | | |
| --- | --- | --- | --- | --- | --- |
|  | AGUS | Atypical glandular cells | M69720 | M69720 |  |
|  | AIS | Adenocarcinoma in situ | M81402 | M81402 | 094 |
|  |  | Adenocarcinoma | M81403 | M81403 | 096 |

^*^ Also including ICD7=171

CIN, cervical intraepithelial neoplasia; SNOMED, Systematized Nomenclature of Medicine(1)

^1^SNOMED classification according to: M. HWW. In: Wagner M MA, Aryel R. , editor. Handbook of Biosurveillance Elsevier 2006. p. 439-52.
