# Supplementary material for: Associations of treated and untreated human papillomavirus infection with preterm delivery and neonatal mortality: A Swedish population-based study
Source: PLoS Med. 2021 May 10;18(5):e1003641. doi: 10.1371/journal.pmed.1003641 (PMC8143418; doi:10.1371/journal.pmed.1003641)
Supplement: S4 Table — (DOC) [file pmed.1003641.s005.doc]

**S4 Table. Outcome definition based on diagnosis codes according to the International Classification of Diseases 10 (ICD-10) in the Swedish Medical Birth Register.**

| ICD 10 codes | Other criteria |
| --- | --- |
| **pPROM** | |
| O42.  O42.0, O42.1, O42.2, O42.9  P01.1 | Gestational age under 263 days^1^, otherwise included in the PROM group |
| **PROM** | |
| O756.6, O75.6A, O75.6B, O75.6X |  |
| **Chorioamnionitis** | |
| O41.1  P02.7  O75.3 |  |
| **Intrapartum fever** | |
| O75.2 |  |
| **Neonatal sepsis** |  |
| P36.0-P36.9  A40, A400, A401, A403, A408, A409  A41,A410, A411, A412, A413, A414, A415, A418, A419 |  |

^1^Induction of labour could start 3 days after pPROM; gestational age <262 days with ICD codes for pPROM was considered correctly diagnosed.

ICD, International Statistical Classification of Diseases and Related Health Problems; MBR, the Swedish Medical Birth Register; pPROM, preterm prelabour rupture of membranes; PROM, prelabour rupture of membranes
