# Supplementary material for: Associations of treated and untreated human papillomavirus infection with preterm delivery and neonatal mortality: A Swedish population-based study
Source: PLoS Med. 2021 May 10;18(5):e1003641. doi: 10.1371/journal.pmed.1003641 (PMC8143418; doi:10.1371/journal.pmed.1003641)
Supplement: S5 Table — (DOC) [file pmed.1003641.s006.doc]

**S5 Table.** **Obstetric and neonatal outcomes in exposure groups, compared to the Reference group, univariable logistic regression analyses.**

|  | Reference  group  (N=  338,109) | HPV infection groups | | | | | | Treated group  (N=23,185) | | | Subsequent CIN2+ group  (N=33,760) | | |
| --- | --- | --- | --- | --- | --- | --- | --- | --- | --- | --- | --- | --- | --- |
|  |  | Cytology (N=11,727) | | | HPV test (N=2,550) | | |  |  |  |  |  |  |
| Outcome | n (%) | n (%) | OR (95% CI) | p-value | n (%) | OR^1^ (95% CI) | p-value^1^ | n (%) | OR (95% CI) | p-value | n (%) | OR (95% CI) | p-value |
| PTD  <37 weeks | 15,661  (4.6) | 692 (5.9) | 1.29  (1.19-1.40) | **<0.001** | 143 (5.6) | 1.23  (1.04-1.46) | **0.016** | 2,106 (9.1) | 2.06  (1.96-2.16) | **<0.001** | 1,736 (5.1) | 1.12  (1.06-1.17) | **<0.001** |
| Early PTD  <34 weeks | 4,221  (1.2) | 221 (1.9) | 1.52  (1.33-1.74) | **<0.001** | 34 (1.3) | 1.05  (0.75-1.48) | 0.77 | 661 (2.9) | 2.32  (2.14-2.52) | **<0.001** | 488 (1.4) | 1.16 (1.06-1.28) | **0.002** |
| Very early PTD  <28 weeks | 820  (0.2) | 55 (0.5) | 1.94  (1.47-2.55) | **<0.001** | 7 (0.3) | 1.03  (0.49-2.18) | 0.93 | 139 (0.6) | 2.48  (2.07-2.97) | **<0.001** | 87 (0.3) | 1.06 (0.85-1.33) | 0.59 |
| Spontaneous PTD | 11,409  (3.4) | 493 (4.2) | 1.26  (1.15-1.38) | **<0.001** | 100 (3.9) | 1.19  (0.97-.1.46) | 0.09 | 1,699 (7.3) | 2.26  (2.15-2.39) | **<0.001** | 1,291 (3.8) | 1.14 (1.07-1.21) | **<0.001** |
| pPROM | 5,110  (1.5) | 232 (2.0) | 1.32  (1.15-1.50) | **<0.001** | 64 (2.5) | 1.62  (1.26-2.08) | **<0.001** | 934 (4.0) | 2.74  (2.55-2.94) | **<0.001** | 521 (1.5) | 1.02 (0.93-1.12) | 0.65 |
| PROM in deliveries at ≥ 37 weeks | 21,906  (6.8) | 828 (7.5) | 1.11  (1.04-1.20) | **0.004** | 251 (10.4) | 1.45  (1.27-1.66) | **<0.001** | 1,772 (8.4) | 1.26  (1.20-1.32) | **<0.001** | 1,719 (5.4) | 0.78 (0.74-0.82) | **<0.001** |
| SGA^2^ | 6,873  (2.0) | 320 (2.7) | 1.35  (1.21-1.52) | **<0.001** | 65 (2.6) | 1.22  (0.95-1.56) | 0.12 | 594 (2.6) | 1.27  (1.16-1.38) | **<0.001** | 715 (2.1) | 1.04 (0.97-1.13) | 0.29 |
| Apgar score <7 at 5 min | 4,165  (1.2) | 191 (1.6) | 1.33  (1.15-1.54) | **<0.001** | 41 (1.6) | 1.23  (0.90-1.68) | 0.19 | 366 (1.6) | 1.29  (1.16-1.43) | **<0.001** | 323 (1.0) | 0.78 (0.69-0.87) | **<0.001** |
| Neonatal mortality | 343  (0.1) | 24 (0.2) | 2.02  (1.34-3.06) | **0.001** | 7  (0.3) | 2.86  (1.35-6.07) | **0.006** | 47 (0.2) | 2.00  (1.47-2.71) | **<0.001** | 29 (0.1) | 0.85 (0.58-1.24) | 0.39 |
| Intrauterine fetal death | 711  (0.2) | 43 (0.4) | 1.75 (1.28-2.38) | **<0.001** | 6  (0.2) | 1.08 (0.48-2.42) | 0.85 | 74 (0.3) | 1.52 (1.20-1.93) | **0.001** | 50 (0.1) | 0.70 (0.53-0.94) | **0.016** |
| Chorioamnionitis | 895  (0.3) | 45 (0.4) | 1.45  (1.08-1.96) | **0.015** | 10 (0.4) | 1.37  (0.73-2.56) | 0.32 | 196 (0.8) | 3.21  (2.75-3.75) | **<0.001** | 74 (0.2) | 0.83 (0.65-1.05) | 0.12 |
| Intrapartum fever | 2,189  (0.6) | 87 (0.7) | 1.15  (0.93-1.42) | 0.21 | 37 (1.5) | 1.96  (1.41-2.72) | **<0.001** | 213 (0.9) | 1.42  (1.24-1.64) | **<0.001** | 133 (0.4) | 0.61 (0.51-0.72) | **<0.001** |
| Neonatal sepsis | 2,508  (0.7) | 97 (0.8) | 1.12  (0.91-1.37) | 0.29 | 14 (0.6) | 0.75  (0.44-1.26) | 0.27 | 300 (1.3) | 1.75  (1.56-1.98) | **<0.001** | 216 (0.6) | 0.86 (0.75-0.99) | **0.036** |

CI, confidence interval; CIN, cervical intraepithelial neoplasia; HPV, human papillomavirus; min, minutes; N, number; OR, odds ratio; pPROM, preterm prelabour rupture of membranes; PROM, prelabour rupture of membranes; PTD, preterm delivery; SGA, small for gestational age

^1^ Analyses compared to Reference group 2007-2016

^2^ Missing data: Reference group n=575, HPV infection (cytology) n=24, HPV infection (HPV test) n=3, Treated n=47, Subsequent CIN2+ n=71
