# Supplementary material for: Associations of treated and untreated human papillomavirus infection with preterm delivery and neonatal mortality: A Swedish population-based study
Source: PLoS Med. 2021 May 10;18(5):e1003641. doi: 10.1371/journal.pmed.1003641 (PMC8143418; doi:10.1371/journal.pmed.1003641)
Supplement: S6 Table — (DOC) [file pmed.1003641.s007.doc]

**S6 Table. Obstetric and neonatal outcomes in the Subsequent CIN2+ group, compared with the Reference group, stratified for interval from delivery to CIN2+ diagnosis, univariable logistic regression analyses.**

|  | Reference group | Subsequent CIN2+ group | | | | | |
| --- | --- | --- | --- | --- | --- | --- | --- |
|  |  | ≤3years after delivery | | | >3 years after delivery ^1^ | | |
| Number | 338,109 | 16,152 | | | 17,608 | | |
| Outcome | n (%) | n (%) | OR (95% CI)^2^ | p-value | n (%) | OR (95% CI)^2^ | p-value |
| PTD, <37 weeks | 15,661 (4.6) | 890 (5.5) | 1.20 (1.12-1.29) | **<0.001** | 846 (4.8) | 1.04 (0.97-1.12) | 0.29 |
| Early PTD, <34 weeks | 4,221 (1.2) | 249 (1.5) | 1.24 (1.09-1.41) | **0.001** | 239 (1.4) | 1.09 (0.96-1.24) | 0.21 |
| Very early PTD, <28 weeks | 820 (0.2) | 45 (0.3) | 1.15 (0.85-1.55) | 0.36 | 42 (0.2) | 0.98 (0.72-1.34) | 0.92 |
| Spontaneous PTD | 11,409 (3.4) | 665 (4.1) | 1.23 (1.14-1.33) | **<0.001** | 626 (3.6) | 1.06 (0.97-1.15) | 0.20 |
| pPROM | 5,110 (1.5) | 288 (1.8) | 1.18 (1.05-1.33) | **0.006** | 233 (1.3) | 0.87 (0.77-1.00) | **0.046** |
| PROM in deliveries at ≥ 37 weeks | 21,906 (6.8) | 927 (6.1) | 0.89 (0.83-0.95) | **0.001** | 792 (4.7) | 0.68 (0.63-0.73) | **<0.001** |
| SGA^3^ | 6,873 (2.0) | 355 (2.2) | 1.08 (0.97-1.21) | 0.15 | 360 (2.1) | 1.01 (0.91-1.12) | 0.90 |
| Apgar score <7 at 5 min | 4,165 (1.2) | 177 (1.1) | 0.89 (0.76-1.03) | 0.13 | 146 (0.8) | 0.67 (0.57-0.79) | **<0.001** |
| Neonatal mortality | 343 (0.1) | 17 (0,1) | 1.04 (0.64-1.69) | 0.88 | 12 (0.1) | 0.67 (0.38-1.19) | 0.18 |
| Intrauterine fetal death | 711 (0.2) | 34 (0.2) | 1.00 (0.71-1.41) | 1.00 | 16 (0.1) | 0.43 (0.26-0.71) | **0.001** |
| Chorioamnionitis | 895 (0.3) | 40 (0.2) | 0.94 (0.68-1.28) | 0.68 | 34 (0.2) | 0.73 (0.52-1.03) | 0.07 |
| Intrapartum fever | 2,189 (0.6) | 83 (0.5) | 0.79 (0.64-0.99) | **0.038** | 50 (0.3) | 0.44 (0.33-0.58) | **<0.001** |
| Neonatal sepsis | 2,508 (0.7) | 124 (0.8) | 1.04 (0.86-1.24) | 0.71 | 92 (0.5) | 0.70 (0.57-0.87) | **0.001** |

CI, confidence interval; CIN, cervical intraepithelial neoplasia; HPV, human papillomavirus; min, minutes; N, number; OR, odds ratio; pPROM, preterm prelabour rupture of membranes; PROM, prelabour rupture of membranes; PTD, preterm delivery; SGA, small for gestational age

^1^ >3-18 years after delivery

^2^ Comparison with Reference group

^3^ Missing data; Reference group n=575, Subsequent CIN2+ ≤3 years after delivery=25, Subsequent CIN2+ >3 years after delivery=46
