# Supplementary material for: Associations of treated and untreated human papillomavirus infection with preterm delivery and neonatal mortality: A Swedish population-based study
Source: PLoS Med. 2021 May 10;18(5):e1003641. doi: 10.1371/journal.pmed.1003641 (PMC8143418; doi:10.1371/journal.pmed.1003641)
Supplement: S7 Table — (DOC) [file pmed.1003641.s008.doc]

**S7 Table. Obstetric and neonatal outcomes in the Treated group, compared to the HPV infection groups and the Subsequent CIN2+group, univariable logistic regression analyses.**

| N | Treated group  (N=  22,711) | HPV infection (cytology) group (N=11,727) | | | Treated group  (N=  14,579) | HPV infection (HPV test) group (N= 2,550) | | | Treated group,  (N=  18,505) | Subsequent CIN2+ group  (N=33,760) | | |
| --- | --- | --- | --- | --- | --- | --- | --- | --- | --- | --- | --- | --- |
| Outcome | n  (%) | n  (%) | OR (95%CI) | p-value | n  (%) | n  (%) | OR^1^  (95% CI) | p-value | n  (%) | n  (%) | OR (95% CI) | p-value |
| PTD  <37 weeks | 2,066  (9.1) | 692 (5.9) | 1.60  (1.46-1.75) | **<0.001** | 1,313  (9.0) | 143 (5.6) | 1.67  (1.39-1.99) | **<0.001** | 1,751  (9.5) | 1,736 (5.1) | 1.93  (1.80-2.07) | **<0.001** |
| Early PTD  <34 weeks | 645  (2.8) | 221 (1.9) | 1.52  (1.30-1.78) | **<0.001** | 416  (2.9) | 34 (1.3) | 2.17 (1.53-3.09) | **<0.001** | 554  (3.0) | 488 (1.4) | 2.10  (1.86-2.38) | **<0.001** |
| Very early PTD  <28 weeks | 136  (0.6) | 55 (0.5) | 1.28  (0.93-1.75) | 0.13 | 98  (0.7) | 7 (0.3) | 2.46  (1.14-5.30) | **0.022** | 118  (0.6) | 87 (0.3) | 2.48  (1.88-3.28) | **<0.001** |
| Spontaneous PTD | 1,669  (7.3) | 493 (4.2) | 1.81  (1.63-2.00) | **<0.001** | 1 038  (7.1) | 100 (3.9) | 1.88  (1.52-2.32) | **<0.001** | 1 421  (7.7) | 1,291 (3.8) | 2.09  (1.94-2.26) | **<0.001** |
| pPROM | 918  (4.0) | 232 (2.0) | 2.09  (1.80-2.42) | **<0.001** | 598  (4.1) | 64 (2.5) | 1.66  (1.28-2.16) | **<0.001** | 779  (4.2) | 521 (1.5) | 2.80  (2.51-3.14) | **<0.001** |
| PROM in deliveries at ≥ 37 weeks | 1,752  (8.5) | 828 (7.5) | 1.14  (1.05-1.25) | **0.002** | 1,208  (9.1) | 251 (10.4) | 0.86  (0.75-0.99) | **0.040** | 1,526  (9.1) | 1 719 (5.4) | 1.77  (1.65-1.90) | **<0.001** |
| SGA^2^ | 587  (2.6) | 320 (2.7) | 0.95  (0.83-1.09) | 0.43 | 383  (2.6) | 65 (2.6) | 1.03  (0.79-1.35) | 0.82 | 523  (2.8) | 715 (2.1) | 1.35  (1.20-1.51) | **<0.001** |
| Apgar score <7 at 5 min | 355  (1.6) | 191 (1.6) | 0.96  (0.80-1.15) | 0.64 | 248  (1.7) | 41 (1.6) | 1.06  (0.76-1.48) | 0.74 | 303  (1.6) | 323 (1.0) | 1.72 (1.47-2.02) | **<0.001** |
| Neonatal mortality | 43  (0.2) | 24 (0.2) | 0.93  (0.56-1.53) | 0.76 | 21  (0.1) | 7 (0.3) | 0.52 (0.22-1.23) | 0.14 | 35  (0.2) | 29 (0.1) | 2.20  (1.35-3.61) | **0.002** |
| Intrauterine fetal death | 71  (0.3) | 43 (0.4) | 0.85 (0.58-1.25) | 0.41 | 51  (0.3) | 6 (0.2) | 1.49 (0.64-3.47) | 0.36 | 58  (0.3) | 50 (0.1) | 2.12 (1.45-3.10) | **<0.001** |
| Chorioamnionitis | 192  (0.8) | 45 (0.4) | 2.21  (1.60-3.07) | **<0.001** | 119  (0.8) | 10 (0.4) | 2.09  (1.10-3.99) | **0.025** | 160  (0.9) | 74 (0.2) | 3.97  (3.01-5.23). | **<0.001** |
| Intrapartum fever | 211  (0.9) | 87 (0.7) | 1.26  (0.98-1.61) | 0.08 | 154  (1.1) | 37 (1.5) | 0.73  (0.51-1.04) | 0.08 | 197  (1.1) | 133 (0.4) | 2.72  (2.18-3.39) | **<0.001** |
| Neonatal sepsis | 294  (1.3) | 97 (0.8) | 1.57  (1.25-1.98) | **<0.001** | 171  (1.2) | 14 (0.5) | 2.15  (1.25-3.71) | **0.006** | 257  (1.4) | 216 (0.6) | 2.19  (1.82-2.62) | **<0.001** |

CI, confidence interval; CIN, cervical intraepithelial neoplasia; HPV, human papillomavirus; min, minutes; N, number; OR, odds ratio; pPROM, preterm prelabour rupture of membranes; PROM, prelabour rupture of membranes; PTD, preterm delivery; SGA, small for gestational age.

^1^ Analyses 2007-2016

^2^ Missing data; Treated N=47, HPV infection (cytology) N=24, HPV infection (HPV test) N=3, Subsequent CIN2+ N=71
