# Supplementary material for: Associations of treated and untreated human papillomavirus infection with preterm delivery and neonatal mortality: A Swedish population-based study
Source: PLoS Med. 2021 May 10;18(5):e1003641. doi: 10.1371/journal.pmed.1003641 (PMC8143418; doi:10.1371/journal.pmed.1003641)
Supplement: S8 Table — (DOC) [file pmed.1003641.s009.doc]

**S8 Table. Outcomes in the HPV infection (cytology) group, compared to the HPV infection (HPV test) group, univariable and multivariable logistic regression analyses, deliveries 2007-2016.**

|  | HPV infection (cytology)  N=6 923 | HPV infection (HPV test)  N=2 550 | Univariable analyses | | Multivariable analyses^1^ | |
| --- | --- | --- | --- | --- | --- | --- |
| Outcome | n (%) | n (%) | OR (95% CI) | p-value | aOR (95% CI) | p-value |
| Preterm delivery <37 weeks | 407 (5.9) | 143 (5.6) | 0.95 (0.78-1.16) | 0.62 | 0.95 (0.77-1.18) | 0.66 |
| Spontaneous PTD | 284 (4.1) | 100 (3.9) | 0.69 (0.76-1.20) | 0.69 | 1.01 (0.78-1.29) | 0.97 |
| pPROM | 132 (1.9) | 64 (2.5) | 1.32 (0.98-1.79) | 0.07 | 1.43 (1.03-1.99) | **0.034** |
| PROM in deliveries at ≥ 37 weeks | 522 (8.0) | 251 (10.4) | 1.34 (1.14-1.57) | **<0.001** | 1.26 (1.06-1.50) | **0.008** |
| SGA | 186 (2.7) | 65 (2.6) | 0.95 (0.71-1.26) | 0.71 | 0.88 (0.65-1.20) | 0.43 |
| Apgar score <7 at 5 min | 123 (1.8) | 41 (1.6) | 0.90 (0.63-1.29) | 0.58 | 0.77 (0.53-1.13) | 0.18 |
| Neonatal mortality | 12 (0.2) | 7 (0.3) | 1.59 (0.62-4.03 | 0.33 | 1.18 (0.42-3.28) | 0.75 |
| Intrauterine fetal death | 31 (0.4) | 6 (0.2) | 0.52 (0.22-1.26) | 0.15 | 0.52 (0.21-1.30) | 0.16 |
| Chorioamnionitis | 25 (0.4) | 10 (0.4) | 1.09 (0.52-2.27) | 0.83 | 0.98 (0.44-2.18) | 0.96 |
| Intrapartum fever | 63 (0.9) | 37 (1.5) | 1.60 (1.07-2.41) | **0.024** | 1.35 (0.87-2.11) | 0.19 |
| Neonatal sepsis | 56 (0.8) | 14 (0.5) | 0.68 (0.38-1.22) | 0.19 | 0.56 (0.30-1.04) | 0.07 |

^1^Adjusted for: year of delivery, maternal age, parity, BMI, marital status, country of birth, infant’s sex, smoking, income, education level, assisted reproduction

aOR, adjusted odds ration; CI, confidence interval; HPV, human papillomavirus; min, minutes; N, number; OR, odds ratio; pPROM, preterm prelabour rupture of membranes; PROM, prelabour rupture of membranes; PTD, preterm delivery; SGA, small for gestational age
