# Supplementary material for: Associations of treated and untreated human papillomavirus infection with preterm delivery and neonatal mortality: A Swedish population-based study
Source: PLoS Med. 2021 May 10;18(5):e1003641. doi: 10.1371/journal.pmed.1003641 (PMC8143418; doi:10.1371/journal.pmed.1003641)
Supplement: Attachment — Submitted filename: Response to reviewers.doc [file pmed.1003641.s010.doc]

Gothenburg March 5^th^ 2021

Dear Editorial board and reviewers,

Thank you for the opportunity to revise our manuscript PMEDICINE-D-20-06245R1, initially submitted with the following title named “Treated and untreated human papillomavirus infection is associated with preterm delivery and neonatal mortality - a Swedish population-based study”.

We have carefully considered your comments and made changes in the manuscript accordingly, which we believe have led to substantial improvement. The comments from the original PLOS Medicine response letter are marked in bold and our responses in non-bold italics. We hope that you find the revised manuscript suitable for publication in PLOS Medicine.

Yours sincerely,

Johanna Wiik & Verena Sengpiel, on behalf of all authors

Johanna Wiik, MD

Dept of Obstetrics and Gynaecology, Sahlgrenska Academy, Gothenburg University, Gothenburg, Sweden, [johanna.wiik@gu.se](mailto:johanna.wiik@gu.se)

And Department of Gynaecology and Obstetrics, Østfold Hospital Trust, Kalnes, Norway

Verena Sengpiel, MD, Associate Professor

Dept of Obstetrics and Gynaecology, Sahlgrenska Academy, Gothenburg University, Gothenburg, Sweden

Dept of Obstetrics and Gynaecology, Sahlgrenska University Hospital, Gothenburg, Sweden

**In your rebuttal letter you should indicate your response to the reviewers’ and editors’ comments, the changes you have made in the manuscript, and include either an excerpt of the revised text or the location (eg: page and line number) where each change can be found. Please submit a clean version of the paper as the main article file; a version with changes marked should be uploaded as a marked up manuscript.**

**Requests from the editors:

1.Title: Please revise your title according to PLOS Medicine’s style. Your title must be nondeclarative and not a question. It should begin with main concept if possible. “Effect of” should be used only if causality can be inferred, i.e., for an RCT. Please place the study design (“A randomized controlled trial,” “A retrospective study,” “A modelling study,” etc.) in the subtitle (ie, after a colon).  We suggest: “Associations of reated and untreated human papillomavirus infection with preterm delivery and neonatal mortality: A Swedish population-based study” or similar.**

*Response 1*

*We have changed the title according to your suggestions:*

*“Associations of treated and untreated human papillomavirus infection with preterm delivery and neonatal mortality: A Swedish population-based study”*

**2. Abstract: Methods and Findings: Please provide some of the key summary demographics of the population.**

*Response 2*

*We have added summary demographics regarding age at delivery, BMI, and frequency of nulliparity for the whole cohort (n=1,044,023) to the Methods and findings section in the abstract.*

*If you should prefer other/more demographic factors, please let us know.*

**3. Abstract: Methods and Findings: Please quantify the main results (with both 95% CIs and p values).**

*Response 3*

*We now quantify the main results with both p-values and 95% CI.*

**4. Abstract: Methods and Findings: In the last sentence of the Abstract Methods and Findings section, please describe the main limitation(s) of the study's methodology.**

*Response 4*

*We have added the following to the Abstract, Methods and Findings;*

*“Limitations of the study include a retrospective design. Since HPV test results only became available in 2007, abnormal cytology was used as a proxy for HPV infection.”*

**5. Abstract: Conclusions:  Please address the study implications without overreaching what can be concluded from the data; the phrase "In this study, we observed ..." may be useful.**

*Response 5*

*Thank you for pointing out our ambiguous formulation, we have changed as follows:*

*“In this study, we found that HPV infection in conjunction with pregnancy was associated with PTD, pPROM, PROM and neonatal mortality. Previous treatment for CIN was associated with even greater risks for these adverse pregnancy outcomes and was also associated with maternal and neonatal infectious complications”.*

**6. Abstract: Conclusions: Please avoid language implying causality (“Treatment for CIN increases risks ruther and seems to confer a risk…”)**

*Response 6*

*Please see under Response 5 above.*

**7. Author summary: At this stage, we ask that you include a short, non-technical Author Summary of your research to make findings accessible to a wide audience that includes both scientists and non-scientists. The Author Summary should immediately follow the Abstract in your revised manuscript. This text is subject to editorial change and should be distinct from the scientific abstract. Please see our author guidelines for more information: [https://journals.plos.org/plosmedicine/s/revising-your-manuscript#loc-author-summary](https://journals.plos.org/plosmedicine/s/revising-your-manuscript#loc-author-summary)**

*Response 7*

*We have added the following text;*

*“Author summary*

*Why was this study done?*

**Treatment of cervical intraepithelial neoplasia (CIN) is associated with an increased risk of preterm delivery (PTD), preterm prelabour rupture of the membranes (pPROM), and neonatal mortality. However, the exact biological mechanism is still unknown.*

**Women with untreated CIN also seem to have an increased risk of PTD. It is unknown whether HPV infection can cause PTD or other adverse obstetric outcomes.*

**Although ascending bacterial infection is an established leading cause of PTD, there is limited knowledge concerning whether PTD after excisional treatment is associated with infectious complications.*

*What did the researchers do and find?*

**This retrospective population-based register study (1999-2016) is, so far, the largest published and the first to demonstrate that HPV infection in conjunction with pregnancy is associated with an increased risk of PTD, pPROM, PROM and neonatal mortality.*

**Treatment for CIN was associated with an even higher risk for these outcomes.*

**This study presents new evidence that previous treatment for CIN is associated with an increased risk of maternal and neonatal infectious complications.*

*What do these findings mean?*

**Although we cannot claim causality due to the register-based study design, our results suggest that pregnancies after treatment for CIN should be regarded as high-risk pregnancies and counselled accordingly.*

** These results support general HPV vaccination programs.”*

**8. Main text: Throughout: Please do not include spaces within brackets of in-text citation (e.g. [2,3]) and please place the brackets before the punctuation (e.g. Page 4, line 2).**

*Response 8*

*This has been changed accordingly.*

**9. Introduction: Please do conclude the Introduction by mentioning your study objective/hypothesis, but this does not need to be in a separate “Objectives” section within the introduction.**

*Response 9*

*We have changed according to your suggestion, please see page 5, line 10-14 and removed the separate Objective section.*

**10. Methods/ figure 1: Is it possible to give additional clarification/explanation of the 600,000+ excluded women (did not fulfil criteria of any group) from Fig 1 flow chart (reasons for exclusion)?**

*Response 10*

*These 600.000+ women had singelton deliveries during the specified time interval but had no exposure qualifying them for inclusion in any of the study groups.*

*So, rather than fulfilling exclusion criteria, these women had no exposure to make them eligible for inclusion in any of the study groups.*

*The criteria for inclusion in the different study groups are specified under Exposures page 7 and in the S2 Table.*

*Since there were many different criteria for inclusion, it is not possible to include them into Figure 1 in a comprehensive manner. However, we tried to clarify Figure 1 by omitting the word “Excluded” from the box and add “inclusion” so that it now reads “Did not fulfil inclusion criteria of any group.”*

**11. Methods:  Please add the following statement, or similar, to the Methods: "This study is reported as per the Strengthening the Reporting of Observational Studies in Epidemiology (STROBE) guideline (S1 Checklist)."**

*Response 11*

*We have added this statement to the first paragraph of the Methods and Material section, page 6 line 4-5.*

**12. Methods: Did your study have a prospective protocol or analysis plan? Please state this (either way) early in the Methods section.

a) If a prospective analysis plan (from your funding proposal, IRB or other ethics committee submission, study protocol, or other planning document written before analyzing the data) was used in designing the study, please include the relevant prospectively written document with your revised manuscript as a Supporting Information file to be published alongside your study, and cite it in the Methods section. A legend for this file should be included at the end of your manuscript.**

**b) If no such document exists, please make sure that the Methods section transparently describes when analyses were planned, and when/why any data-driven changes to analyses took place.**

**c) In either case, changes in the analysis-- including those made in response to peer review comments-- should be identified as such in the Methods section of the paper, with rationale.**

*Response 12*

*This study was prospectively planned and received approval from Swedish Ethical Review Authority in 2015, the dataset was finally received in 2019 and analyses conducted until November 2020. While we did not publish any analysis plan, the overall exposure, outcomes and confounders and analyses were specified in the ethical application and based on hypothesis drawn from previous studies. This ethical application also includes plans of other upcoming studies and therefore we would prefer not to publish it online.*

*After obtaining and reviewing the content of the dataset, but before starting the analyses, the final definition of study groups, outcome and study period was decided based on the available data and quality of the dataset. During the analysis process, we decided to*

*add stratified analyses to the analysis plan.*

*Please see added text page 6 line 5-12.*

**13. Results: Please provide more quantitative description of the background differences in study groups (age, parity, smoking).**

*Response 13*

*All descriptive information is presented in Table 1. We have now emphasized the major differences in the text as well, page 10, line 19-30;*

*“The study groups differed, for example regarding age, parity and smoking. Of the references 10 % (n=32,457 women) were older than 35 years and similarly 10 % in the Subsequent CIN2+ group (n=3,492) and 12% in the HPV infection (cytology) group (n=1,366) were older than 35 years, while this was more frequent in the HPV infection (HPV test) group (n=429, 17%), and the Treated group (n=4,755, 21%). Nulliparity was most frequent in the Treated group (n=13,425, 58%) and HPV infection groups (cytology; n=6,479, 55% and HPV test; n=1,481, 58%) and less frequent in the Reference group (n=163,533, 48%) and Subsequent CIN2+ group (n=12,730, 38%). Smoking during pregnancy was less common in the Reference group (n=15,365, 4.5%) than the exposure groups; HPV infection (cytology) (n=1,471, 12.5%), HPV infection (HPV test) (n=177, 6,9%), Treated group (n=2,811, 12.1%) and Subsequent CIN2+ group (n=5,895, 17,5%).”*

*While adding the descriptive information to the text, we realized that we included an older version of Table 1. We apologize for this. The data in Table 1 is now corrected. Only Table 1 had incorrect data, results presented in the other tables are correct.*

**14. Results: For all results presented in the text, please provide both the 95% CIs and p values.**

*Response 14*

*We have added the 95% CIs and p-values in the text accordingly throughout the manuscript.*

**15. Results: “Small for gestational age and Apgar score” section: please clarify which groups are meant by “The groups did not differ…” in the second sentence of this paragraph.**

*Response 15*

*We have changed to:”There was no difference between the Reference group and any of the exposure groups in regard to SGA risk (Table 2)”, page 17, line 12-13.*

**16. Results: “HPV infection groups” section: please report the result for this association “Except for a higher risk of pPROM and PROM in the HPV infection (HPV test) group” and specify the comparison group.**

*Response 16*

*We have changed as follows:”Except for a higher risk of pPROM (OR 1.43 95% CI 1.03-1.99, p=0.034) and PROM (OR 1.26, 95% CI 1.06-1.50, p=0.008) in the HPV infection (HPV test) group compared to the HPV infection (cytology) group with no HPV test, no significant differences in outcomes were found between the HPV infection groups (S8 Table).”, page 20, line 18-21.*

**17. Discussion: Please present and organize the Discussion as follows: a short, clear summary of the article's findings; what the study adds to existing research and where and why the results may differ from previous research; strengths and limitations of the study; implications and next steps for research, clinical practice, and/or public policy; one-paragraph conclusion.**

*Response 17*

*We have in the Discussion added the headlines Main results, Comparison with previous studies and interpretation, Strengths and Limitations, Implications and next step for research. The “Comparison with previous studies and interpretation” section comprises the subheadings Treatment for CIN and obstetric and neonatal outcome and HPV infection and obstetric and neonatal outcomes.*

*We moved the following text to the “Strengths and Limitations” section, page 22 line 1-4, “To the best of our knowledge, this is the largest study examining the impact of HPV infection in conjunction with pregnancy and treatment for CIN on obstetric and neonatal outcome. It comprises an entire population and compares the largest number of deliveries before and after treatment for CIN in the same woman published so far”. After this text we describe limitations of the study; page 22, line 5-28.*

*We have moved and rephrased the following text to the “Implications and next step for research” section, page 22, line 31-34 and page 23 line 1-3, “The use of HPV tests in cervical cancer screening has increased during the last decade, facilitating large population-based analysis of the effects of HPV infection and pregnancy outcomes in the near future. Our results support general HPV vaccination programs and future studies in vaccinated cohorts will ascertain whether PTD and neonatal mortality decrease. Additional experimental studies are also needed to establish the causal pathways linking HPV infection and treatment for CIN to PTD and other adverse obstetric outcomes. This may be a key to understand the biological mechanism leading to PTD.*

*We have also added the sentence “We suggest that pregnancies after treatment for CIN should be regarded as high-risk pregnancies” to this paragraph.*

**18. References: Please double check the formatting (such as use of italics- e.g. BMJ format in ref 8 and 15).**

**Please provide an English translation for ref 26, 28, 29, 30.**

*Response 18*

*We have checked the references as suggested.*

*We have added English translations for the references named above.*

**19. Table 2, Table 3, Table, 4, and Table 5: Please present actual p values rather than representing with asterisks.  Please provide the unadjusted results in addition to the adjusted analyses (these may be in a supporting information table, if desired).**

*Response 19*

*We have removed the asterisks and added p-values. We present two decimals in the p-value if not significant and three decimals if significant or close to significant.*

*Unadjusted and adjusted analyses are now presented in Table 5 and unadjusted analyses for the comparisons in Table 2-4 have been added to the supporting information in S5-S7 Tables. We added a reference to the tables presenting unadjusted analyses in the result section; “Comparisons presented below for different obstetrical outcomes are adjusted logistic regression analyses, unadjusted analyses are presented in S5-7 Tables”, page 13, line 1-2.*

***20. Figures 2 and 3: Please include the p values with the arrows, if possible.***

**As mentioned for the Tables, the unadjusted analyses should be included, whether in the main text or supporting information.**

*Response 20*

*The information has been added*

**21. S5 Table: Please provide p values for each comparison, rather than indicating p values with asterisks.  Please provide the unadjusted analyses.**

*Response 21*

*P-values have been added. We present two decimals in the p-value if not significant and three decimals if significant or close to significant. Unadjusted analyses have been added, please see S8 Table.*

**22. STROBE checklist: Thank you for including the checklist.  It would be appropriate to refer to locations in the text for each item with an additional column on the right-hand side of the table- When completing the checklist, please use section and paragraph numbers, rather than page numbers.**

*Response 22*

*We have changed the STROBE checklist according to your suggestions.*


**Comments from the reviewers:**

**Reviewer #1: This retrospective population-based register study of women with singleton deliveries registered in the Swedish Medical Birth Register 1999-2016, aims to investigate whether HPV infection in conjunction with pregnancy, as well as previous treatment for CIN, is associated with an increased risk of PTD and other adverse obstetric and neonatal outcomes.

Comments:
There are some typos in the text that need rectifying.
The STROBE checklist is suitably included in the supplementary material.

Descriptive data are appropriately presented, and the comparison of exposure groups by logistic regression (adjusted for socioeconomic and health-related confounders) is a technically valid and robust approach.

Are there other factors that may be associated with PTD that can be included as potential confounders within the models?  Alcohol or drug abuse, for instance?**

*Reviewer#1*

*Response*

*This is good suggestion. However, this study was limited to the data available in the Swedish registers. Therefore, we had no possibility to adjust for alcohol or drug abuse.*

**Table 1 is very informative.  Did the authors complete any statistical testing to explore if differences between groups are significant or not?**

*Response*

*We decided to use prespecified potential confounding factors in the adjusted analyses, based on previous research. They are included in Table 1 together with further descriptive variables. The exposure characteristics used for defining the groups naturally resulted in uneven distribution for many of these factors. Since we decided to adjust for these factors a priori, we did not perform any formal significance testing. This is described in statistical analyses page 9 line 20-21; “The multivariable analyses were adjusted for potential confounding factors, including background variables with an uneven distribution between the groups”*

**Table 2 is an efficient and effective way to communicate the main results.
Perhaps more caution should be applied when drawing inferences from the paired analysis.  These results might not be generalisable due to the specific nature of the cohort analysed. 
For instance:  Might multiple births be affecting the outcomes?  Or, the effect of having treatment after at least one prior delivery?  Are there other potential intra-woman confounders in this analysis (e.g age at delivery older after treatment, although it is noted that maternal age is included as a covariate in the model)?**

*Response*

*We are grateful to the reviewer for pointing out this issue. In the Table 5 we presented unadjusted results, not adjusted analyses as we falsely indicated in the text under the table. We have corrected this error. We now also present adjusted paired analyses in the manuscript´s text and in Table 5, together with the unadjusted paired analyses in Table 5. In these analyses we adjusted for smoking, BMI, assisted reproduction, marital status and infant´s sex. We did not adjust for socioeconomic variables (income, education or birth place) since we compared outcomes in the same individual. As a result of the paired analyses before and after treatment, the women are always older and have higher parity in the birth after compared to the birth before treatment. Therefore, it was not possible to adequately adjust for parity and maternal age. We have added a sentence about this under limitations in the discussion section, page 22, line 27-28; “For the paired analyses maternal age and parity were not possible to adjust for and these results must thus be interpreted with caution”.*

**The main limitations have been acknowledged, including the fact that this in an observational study (and therefore, causality cannot be inferred), the potential for confounding, and the possibility of misclassification. Overall, this is a thorough study looking at different subgroups and their outcomes over a large cohort and incorporating a good use of visualisation in the figures which helps to clearly communicate the research findings.**

*Response*

*Thank you for your appraisal of our study.*

**Reviewer #2: This is a large population-based study that investigates in detail the association between HPV infection, CIN or CIN treatment and PTB. The authors have put a lot of effort and have conducted a comprehensive analysis by including all possible comparison groups (women with positive HPV testing prior to delivery; women with abnormal cytology prior to delivery; women treated for CIN3 prior to delivery; women with CIN2+ after delivery; women with no history of HPV/CIN prior to delivery) and performing all possible comparison combinations including self-matching. The authors very nicely associate their findings with previous studies, and suggest potential mechanism of PTB based on their findings. Although the main findings of this study have been suggested by other studies, findings were conflicting or not conclusive before as the authors describe in the manuscript. This is the largest and most comprehensive study so far trying to cast light on the long-standing debate whether HPV infection per se increases the risk of PTB, and therefore I highly recommend it for publication in PLoS Medicine.**

**I have only some minor comments to the authors:
-Line 34: Replace "outcome" with "outcomes"**

*Reviewer#2*

*Response*

*Thank you for your appraisal of our study.*

*We have changed “outcome” to “outcomes” throughout the manuscript.*

**-In the HPV infection group, I do not understand what the authors mean by "between six months prior to conception and delivery". I think that they mean "within six months prior to conception or during pregnancy".I suggest rephrasing throughout the text.**

*Response*

*Thank you for pointing out the need for clarification. We have changed to; “Women with presumed HPV infection during pregnancy, based on abnormal cervical cytology or positive HPV test recorded within six months prior to conception or during pregnancy”, page 7, line 5-7. We have also changed this description in S2 Table and in the Abstract.*

**-Additionally, in abstract the various comparison groups are not very clear. I understand that there is a word limitation, but if possible, authors could describe the different groups in more details, especially the timing of the abnormal test/intervention. By reading the abstract only, it is not very clear for example whether the HPV positivity group includes women with HPV prior to pregnancy, during pregnancy, or both.**

*Response*

*Thank you for pointing out the difficulties in the understanding. We have changed to; “Women with a history of exclusively normal cytology (n=338,109) were compared to women with positive HPV-tests (n=2,550) or abnormal cytology (n=11,727) within six months prior to conception or during the pregnancy, women treated for CIN3 before delivery (n=23,185) and with CIN2+ diagnosed after delivery (n=33,760)”, in the Abstract.*

**-Page 7, lines 16-17: I suggest adding citations for the statistical software that the authors have used.**

*Response*

*We have changed as follows;*

*“Analyses were performed using “R” data analysis tool (version 4.0.0, The R Foundation for Statistical Computing, Vienna, Austria,* [*https://www.r-project.org/*](https://www.r-project.org/)*) and SPSS software (version 26.0, IBM,* [*https://www.ibm.com/analytics/spss-statistics-software*](https://www.ibm.com/analytics/spss-statistics-software)*)”, page 8 line 22-24.*

**-Page 7, line 32: Replace "Subsequent" with "subsequent"**

*Response*

*We have used a Capital letter in all group names to clarify that the expression is just a part of the group name and therefore we would prefer to keep it as it is.*

**Reviewer #3: This is a well-written substantive retrospective paper based on over 1 million births in Sweden.
1. p.5  Line 29. Women with exclusively normal cytology. Does that mean that they had documented negative HPV tests? I ask because if they did not, how do you know that they were not also HPV positive which would put them in the comparison group.**

**When the term Lifetime is used -what does that mean? Is it the time prior to delivery?**

*Reviewer #3,*

*Response 1*

*Thank you for pointing out the need for clarification.*

*The Reference group consists of women who have participated in the Swedish cervical screening program and have no recorded abnormal cervical cytology sample in NKCx.*

*This is specified in the S3 Table “Definition of study groups”.*

*By using the term “lifetime” we meant that until the end of the study period (2016) there were no abnormal tests. We agree that this description was not correct and hard to understand as some women were still young at the end of the study period. We have removed the expression “lifetime” in the abstract (page 2 line 17) and in the main text and changed to “until the end of the study period” Page 7 line 2.*

*Since HPV testing has been introduced in broad scale only recently and test results are available in the dataset first from 2007, we decided not to use a negative HPV test as a criterium for the reference group. It is correct that women in the reference group might have had an undetected HPV infection. As most HPV-infections resolve within a year that would have been the case also if women had been tested for HPV every third or fifth year. Thereby, using only results from screening test for HPV-exposure of course represents an underestimation of the full exposure of HPV. However, if some women with HPV infection wrongly were included into the reference group, such misclassification would have attenuated the associations we found. We have added a sentence about this in the discussion section under the “Strengths and limitations” section, page22 line 22-25;”We did not require a negative HPV test in the Reference group and some women might have had an undetected HPV infection. However, such misclassification would have attenuated the associations found between the exposure groups compared to the Reference group”.*

**2. p.6, line 13: It is possible that women who have CIN2+ at a young age can clear spontaneously. Did you exclude because they got categorized into the other exposure groups?**

*Response 2*

*Thank you for pointing out the need for clarification. Both CIN2 and CIN3 can resolve spontaneously. Non-pregnant women with CIN3 have always been treated in Sweden. Traditionally this applies also for CIN2. However, in recent years young women with CIN2 have sometimes been followed conservatively instead of being treated. On the other hand, sometimes persistent CIN1 is also treated.*

*Women with CIN3 were included in the Treated group. As we did not want any treated women to be included in any of the other exposure groups, women with CIN2+ at any time or with CIN1 more than once before the included delivery were excluded from the other exposure groups. We have tried to clarify this by adding “from these groups” to the following sentence; “In order to avoid women previously treated for CIN being included in the HPV infection groups or the Subsequent CIN2+ group, women with histologically diagnosed CIN1 more than once or with CIN2+ before the pertinent delivery were excluded from these groups”, page 7, line 17-19;*

**3. How did you account for parity? Were women who had multiple births counted as one person or was each birth counted? This could mean a women with multiple preterm births could be counted multiple times with the same characteristics over and over again.**

*Response 3*

*Only one delivery from the same woman was included into the different groups, please see page 8 line 28-32 and page 9, line 1-2;*

*“If a woman had several deliveries that fulfilled the criteria for inclusion in a group, only one delivery was included. A random delivery in the Reference group was compared to the first eligible delivery after treatment in the Treated group and the last eligible delivery in the HPV infection groups (cytology and HPV test) and the last delivery before diagnosis of CIN2+ in the Subsequent CIN2+ group. The first eligible delivery after treatment in the Treated group was compared to the last delivery in a woman included in the other exposure groups.”*

**4. P2, line 31: With the high prevalence of HPV infection in anyone who has ever been sexually active, it may be more appropriate to say, "Women with detectable HPV infection..."**

*Response 4*

*Thank you for your suggestion. The Swedish cervical cancer screening program, that our dataset is based on, has of course not been able to detect all HPV infections in the population during the study period, see also Response 1. If all detectable HPV infections were to be detected one have to repeat HPV-test/Cytology more frequently. Our data is based on what have been detected but not on what is detectable. Therefore, we would prefer not to add detectable in the Abstract. However, we have now added following sentence to the “Strengths ad Limitations” section in the Discussion*; “*To begin with, relying on positive screening tests implies an obvious underestimation of the true incidence and prevalence as many infections can occur and resolve between testing”,, page 22, line 10-12.*
