# Supplementary material for: Associations of treated and untreated human papillomavirus infection with preterm delivery and neonatal mortality: A Swedish population-based study
Source: PLoS Med. 2021 May 10;18(5):e1003641. doi: 10.1371/journal.pmed.1003641 (PMC8143418; doi:10.1371/journal.pmed.1003641)
Supplement: Attachment — Submitted filename: Rebuttal letter .doc [file pmed.1003641.s011.doc]

Gothenburg April 24^th^ 2021

Dear Editorial board,

Thank you for considering to publish our manuscript "Associations of treated and untreated human papillomavirus infection with preterm delivery and neonatal mortality: A Swedish population-based study" (PMEDICINE-D-20-06245R2) in PLOS Medicine, initially submitted with the following title named “Treated and untreated human papillomavirus infection is associated with preterm delivery and neonatal mortality - a Swedish population-based study” (PMEDICINE-D-20-06245R1).

We have carefully considered your comments and made changes in the manuscript accordingly. The comments from the original PLOS Medicine response letter are marked in bold and our responses in non-bold italics. We hope that you find the revised manuscript suitable for publication in PLOS Medicine.

Yours sincerely,

Johanna Wiik & Verena Sengpiel, on behalf of all authors

Johanna Wiik, MD

Dept of Obstetrics and Gynaecology, Sahlgrenska Academy, Gothenburg University, Gothenburg, Sweden, [johanna.wiik@gu.se](mailto:johanna.wiik@gu.se)

And Department of Gynaecology and Obstetrics, Østfold Hospital Trust, Kalnes, Norway

Verena Sengpiel, MD, Associate Professor

Dept of Obstetrics and Gynaecology, Sahlgrenska Academy, Gothenburg University, Gothenburg, Sweden

Dept of Obstetrics and Gynaecology, Sahlgrenska University Hospital, Gothenburg, Sweden

------------------------------------------------------------
**Requests from Editors:

1. Abstract: Methods and Findings: Please clarify if the OR reported in the text here are the adjusted ORs.**

*Response 1*

*We have now added “adjusted” before odds ratio, and changed OR to aOR, page 2 line 59. We have also changed this in the text and tables throughout the manuscript when relevant.*

*All line numbers mentioned here and below refer to the “Clean version of the Manuscript”.*

**2. Abstract: Methods and Findings: Page 3, Line 1-2: We suggest revising the limitations sentence as “Limitations of the study include the retrospective design and the fact that because HPV test results only became available in 2007, abnormal cytology was used as a proxy for HPV infection.”**

*Response 2*

*We have revised the sentence as suggested, page 3, line 70-72.*

**3. Author summary: What did the researchers do and find? In the first bullet point of this section, we suggest removing “...is, so far, the largest published and the first to demonstrate…”**

*Response 3*

*We have now removed this as suggested and changed as follows; “This retrospective population-based register study (1999-2016) found that HPV infection in conjunction with pregnancy is associated with an increased risk of PTD, pPROM, PROM and neonatal mortality”, page 3, line 91-93.*  **4. Author summary: What do these findings mean? We suggest providing some clarification on the final bullet point in this section, for example “These results support the idea that strategies to mitigate HPV infection, such as vaccination programs, may be beneficial for maternal and neonatal pregnancy outcomes.” or similar.**

*Response 4*

*We have changed according to your suggestion, page 3, line 102-103.*

**5. Methods: Page 6: Lines 6-8:  Please clarify this sentence to: “We did not publish the analysis plan but the overall exposure, outcomes, confounders and analyses were planned in 2015 by the research team based on hypotheses drawn from previous studies.”**

*Response 5*

*Thank you for pointing out the need for clarification. We have rephrased as suggested, page 6, line 179-181.*  **6. Methods: Page 6, line 19: Please change to “30.2 years”**

*Response 6*
*This has now been corrected.*

**7. Results: Page 17, line 4: We suggest replacing “non-significant” with “not statistically significant” or similar.**

*Response 7*

*This has now been changed to “The associations for the HPV infection (cytology) group were oriented in the same direction but were not statistically significant (Table 2)”, page 16, line 444-446.*

**8. Discussion: Page 21: Lines 25-26: We suggest revising to: “However, our results suggest that HPV infection might be an important factor.”**

*Response 8*
*We have revised the sentence as suggested, page 21, line 596-597.*

**9. Discussion: Page 22: Line 1-2: Please revise to: “To the best of our knowledge, this is the largest study to date examining the associations of HPV infection in conjunction with pregnancy and treatment for CIN with obstetric and neonatal outcomes.”  Please also temper the second sentence (compares the largest number…) at lines 3-4 with “to the best of our knowledge” or similar.**

*Response 9*

*We have changed as follows; “To the best of our knowledge, this is the largest study to date examining the associations of HPV infection in conjunction with pregnancy and treatment for CIN with obstetric and neonatal outcomes. It comprises an entire population and we believe it compares the largest number of deliveries before and after treatment for CIN in the same woman published so far, page 21, line 606-609.*

**10. Discussion: Page 22, Line 11: We suggest changing “...implies an obvious underestimation…” to “...may lead to an underestimation of the true incidence…” or similar.**

*Response 10*

*We have changed as suggested and the sentence now reads as follows; “To begin with, relying on positive screening tests may lead to an underestimation of the true incidence and prevalence as many infections can occur and resolve between testing”, page 21, line 615-617.* **11. Discussion: Page 22, Line 27-28: Please revise to “For the paired analyses it was not possible to adjust for maternal age and parity and these results must thus be interpreted with caution.”**

*Response 11*

*We have revised the sentence as suggested, page 22, line 632-633.*

**12. Discussion: conclusions: Page 23: Line 9-11: Please revise to avoid causal implications: “Treatment for CIN was associated with further increased risk of PTD, pPROM, PROM and neonatal mortality and was associated with increased risk of maternal and neonatal infectious complications. These results suggest it may be beneficial to regard pregnancy after treatment for CIN as high-risk pregnancies and counselled accordingly.”**

*Response 12*

*We have rephrased Conclusion, and it now reads as follows;*

*“This large population-based study demonstrates that women with HPV infection in conjunction with pregnancy have an increased risk of PTD, pPROM, PROM and neonatal mortality. Treatment for CIN was associated with further increased risk of PTD and pPROM and was also associated with PROM, neonatal mortality and with an increased risk of maternal and neonatal infectious complications. These results suggest that pregnancies after treatment for CIN should be regarded as high-risk pregnancies and counselled accordingly. Furthermore, our results support general HPV vaccination programs”.*

**13. References: Please check journal name abbreviations (Ref 2 should be J Natl Cancer Inst, Ref 3 should be Eur J Cancer). Please remove the italics from ref 17, and 27.  Please use the "Vancouver" style for reference formatting, and see our website for other reference guidelines [https://journals.plos.org/plosmedicine/s/submission-guidelines#loc-references](https://journals.plos.org/plosmedicine/s/submission-guidelines#loc-references)**

*Response 13*

*We have checked journal name abbreviations and changed the references as suggested.*  **14. Figure 2.  Please give an overall title for Figure 2, noting the differences between panels a and b.**

*Response 14*

*We have changed to “Fig 2. Risk of preterm delivery (2a) and spontaneous preterm delivery (2b), adjusted logistic regression”, page 13, line 375-376.*  **15. Figure 4 and Figure 5. Please provide the numbers in addition to percentages.**

*Response 15*

*Thank you very much for this suggestion. We have added the numbers in Figures 4 and 5. While doing this we realized that the % with PTD that had chorioamnionitis/neonatal sepsis in Figure 5 was incorrect. It reflected the % of women with only PTD and chorioamnionitis (not including those with neonatal sepsis). This has now been corrected.*

**16. Table 5: Please note that the line numbers are overlapping with the right-side column for the last 5 rows.**

*Response 16*

*We have now corrected this. We have also changed to continuous line numbers throughout the manuscript. Our responses refer to line numbers in the clean version of the Manuscript.*

**17. Supporting information: If possible, please include each item as a separate file.**

*Response 17*

*We have now separated the tables S1-8 and the STROBE checklist into 9 different Supplemental files.*
 **18. S8 Table: It is not clear what part of the table this reference is supporting (1. M. HWW. In: Wagner M MA, Aryel R. , editor. Handbook of Biosurveillance Elsevier 2006. p. 439-52.)**

*Response 18
It refers to an explanation of the SNOMED classification system, S3 Table. Please see Supporting information S3 Table. After having separated the Supporting tables into different files and adding an explanation below the table, we hope that this is clear.*

Any attachments provided with reviews can be seen via the following link:

__________________________________________________
In compliance with data protection regulations, you may request that we remove your personal registration details at any time.  (Use the following URL: <https://www.editorialmanager.com/pmedicine/login.asp?a=r>). Please contact the publication office if you have any questions.
